# Dynamic barefoot plantar pressure in gait and foot type biomechanics

**DOI:** 10.1186/1757-1146-7-S1-A77

**Published:** 2014-04-08

**Authors:** Jinsup Song, Howard J Hillstrom, Michael Neary, Kersti Choe, William Brechue, Rebecca A Zifchock, Steve Svoboda, Jim Furmato, Mandy Gibbons, Ibadete Thaqi, Jocelyn Hafer, Siobhan Mangan, Stephen Bartalini, Marian T Hannan

**Affiliations:** 1 Temple University School of Podiatric Medicine, Philadelphia, Pennsylvania, USA; 2 Hospital for Special Surgery, New York, New York, USA; 3 United States Military Academy, West Point, New York, USA; 4 Hebrew Senior Life, Harvard Medical School, Boston, USA

## 

Song et al demonstrated that healthy subjects with planus and neutral foot type exhibited a distinguishable foot posture and dynamic foot function [1]. However, such a relationship has not been demonstrated in a large sample study.

Foot structure was categorized into one of three foot types (cavus, neutral, and planus) based on the standing arch height index (AHI) in 1,054 incoming cadets at the US Military Academy (172 female, 18.5±1.1 years, 24.5±3.0 kg/m^2^) [2]. Five trials of barefoot dynamic planar pressure were obtained for each foot with the Novel emed-x (novel GmbH, Munich) using the two-step method for walking data acquisition. The Center of Pressure Excursion Index (CPEI, %) and the peak pressure (PP, in kiloPascal) were calculated for each trial. Analysis of Variance was performed across the foot type groups on the left foot.

The cavus group exhibited the largest CPEI while the planus group demonstrated the smallest CPEI. The neutral group demonstrated the lowest peak pressure, which was significantly lower than the planus group. Results of this study provide additional evidence which support the link between the dynamic plantar pressure in gait and foot type biomechanics.

**Table 1:** The mean Center of Pressure Excursion Index and the Peak Pressure are shown for three foot type groups. The analysis was limited to left foot only.

	Cavus	Neutral	Planus	P-value
N (female)	53 (5)	184 (29)	711 (121)	

CPEI (%)	23.07 ± 7.46	21.01 ± 6.53	20.39 ± 6.82	0.0168 ^a^

PP (kN)	578.5 ± 140.6	552.8 ± 139.2	600.4 ± 168.2	<0.0001 ^c^

A significant difference (P<0.05) was observed between ^a^ the cavus and planus foot types and ^c^ between neutral and planus foot types.
